# Crystal structure of *cis*-bis­[4-phenyl-2-(1,2,3,4-tetra­hydro­naphthalen-1-yl­idene)hydrazinecarbo­thio­amidato-κ^2^ *N* ^1^,*S*]nickel(II) monohydrate tetra­hydro­furan disolvate

**DOI:** 10.1107/S1600536814016079

**Published:** 2014-07-19

**Authors:** Adriano Bof de Oliveira, Bárbara Regina Santos Feitosa, Christian Näther, Inke Jess

**Affiliations:** a Departamento de Química, Universidade Federal de Sergipe, Av. Marechal Rondon s/n, Campus, 49100-000 São Cristóvão–SE, Brazil; b Institut für Anorganische Chemie, Christian-Albrechts-Universität zu Kiel, Max-Eyth Strasse 2, D-24118 Kiel, Germany

**Keywords:** thio­semicarbazone complex, anagostic inter­actions, crystal structure

## Abstract

The reaction of Ni^II^ acetate tetra­hydrate with the ligand 4-phenyl-2-(1,2,3,4-tetra­hydro­naphthalen-1-yl­idene)hydrazinecarbo­thio­amide in a 2:1 molar ratio yielded the title compound, [Ni(C_16_H_16_N_3_S)_2_]·2C_4_H_8_O·H_2_O. The deprotonated ligands act as *N*,*S*-donors, forming five-membered metallacycles with the metal ion exhibiting a *cis* coordination mode unusual for thio­semicarbazone complexes. The Ni^II^ ion is four-coordinated in a tetra­hedrally distorted square-planar geometry. *Trans*-arranged anagostic C—H⋯Ni inter­actions are observed. In the crystal, the complex mol­ecules are linked by water mol­ecules through N—H⋯O and O—H⋯S hydrogen-bonding inter­actions into centrosymmetric dimers stacked along the *c* axis, forming rings of graph-set *R*_4_^4^(12). Classical O—H⋯O hydrogen bonds involving the water and tetra­hydro­furan solvent mol­ecules as well as weak C—H⋯π inter­actions are also present.

## Abstract 2

Crystal structure of a Ni^II^–thio­semicarbazone complex showing an unusual *cis* arrangement of the *N*,*S*-donor ligands and anagostic C—H⋯Ni inter­actions.

## Chemical context   

Thio­semicarbazone ligands are *N*,*S*-donors that show a wide range of coordination modes (Lobana *et al.*, 2009 ▶). As a part of our ongoing project on the synthesis and structures of thio­semicarbazone derivatives and their metal complexes, the crystal structure of an Ni^II^ complex of 2-(1,2,3,4-tetra­hydro­naphthalen-1-yl­idene)-4-phenyl-hydrazinecarbo­thio­amide is reported. The crystal structure of the free ligand was published recently by our group (de Oliveira *et al.*, 2014 ▶), but one of the first reports on the synthesis of thio­semicarbazone deriv­a­tives was done by Freund & Schander (1902 ▶). The complex shows a *cis* coordination mode, which is unusual for this ligands, and two *trans*-arranged anagostic inter­actions between C—H groups and the metal ion are also observed. These inter­actions are typical for several complexes with catalytic applications (Brookhart *et al.*, 2007 ▶).
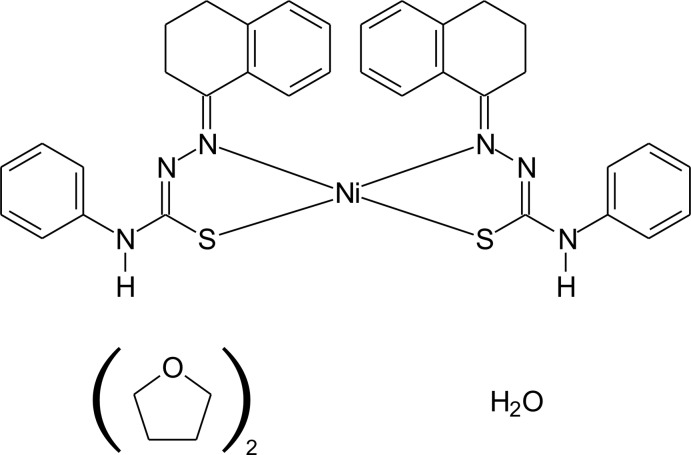


## Structural commentary   

**Figure 1:**
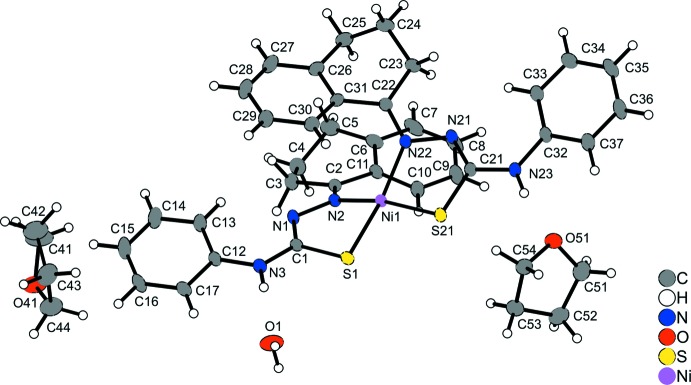
The mol­ecular structure of the title compound with displacement ellipsoids drawn at the 40% probability level.

**Figure 2:**
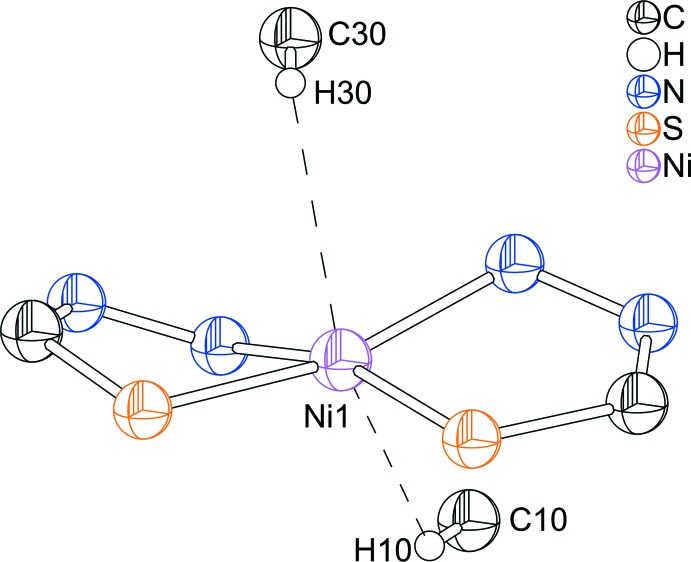
Coordination environment of the metal ion showing the C—H⋯*M* anagostic inter­actions (dashed lines).

In the crystal structure of the title compound, the Ni^II^ cation is four-coordinated by two crystallographically independent deprotonated ligands into discrete complexes that are located in general positions (Fig. 1 ▶). The metal displays a remarkable tetra­hedrally distorted square-planar coordination geometry (maximum displacement 0.5049 (13) Å for atom N2) with the ligands showing an uncommon *cis N* ^1^,*S*-coordination mode. The values of the Ni—N and N—S bond lengths (Table 1 ▶) and N2—Ni1—S21 and N22—Ni1—S1 bond angles [164.04 (5) and 162.63 (4)°, respectively] confirm the distortion from the ideal coordination geometry. In the complex mol­ecule significant structural changes of the N–N–C–S fragment are observed. For the non-coordinating2-(1,2,3,4-tetra­hydro­naphthalen-1-yl­idene)-4-phenyl-hydrazinecarbo­thio­amide ligand, the N—N, N—C and C—S bond lengths amount to 1.385 (2), 1.364 (2) and 1.677 (2) Å. These lengths indicate the double-bond character of the N=N and C=S bonds, and the single-bond character of the N–C bond (de Oliveira *et al.*, 2014 ▶). In contrast, in the title complex the acidic hydrogen of the hydrazine fragment is removed and the negative charge is delocalized over the N–N–C–S fragment. Therefore, the N—N, N—C and C—S bond lengths amount to 1.405 (2), 1.304 (2) and 1.757 (2) Å respectively in one ligand and 1.401 (2), 1.298 (3) and 1.761 (2) Å in the other. The N—C bond lengths indicate a considerable double-bond character, while the N—N and C—S bond distances are consistent with an increased single-bond character. It is worth noting that two *trans*-arranged anagostic inter­actions between aromatic C—H groups and the metal ion are observed (Fig. 2 ▶). For a three-centre–two-electron *M*⋯H—C agostic inter­action, the *M*⋯H distance should range between 1.8 and 2.3 Å and the *M*⋯H—C angle should range between 90 and 140°. For an anagostic inter­action these values should range from 2.3 to 2.9 Å and from 110 to 170°, respectively (Brookhart *et al.*, 2007 ▶). The title complex shows Ni1⋯H30 and Ni1⋯H10 contacts of 2.61 and 2.45 Å [both values are shorter than the sum of the van der Waals radii for Ni (1.63 Å; Bondi, 1964 ▶) and H (1.10 Å; Rowland & Taylor, 1996 ▶)], and C30—H30—Ni1 and C10—H10—Ni1 angles of 118 and 121°, in agreement with the presence of anagostic inter­actions.

## Supra­molecular features   

**Figure 3:**
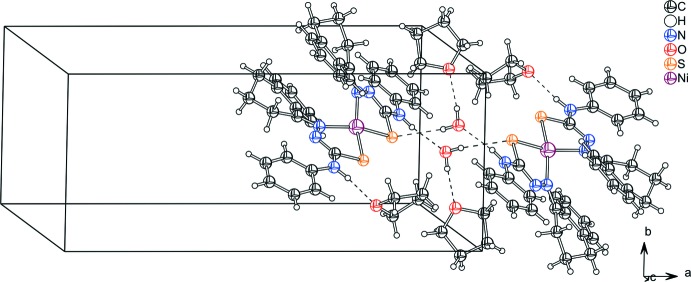
Mol­ecules of the title compound connected through inversion centres *via* pairs of N—H⋯O and O—H⋯S inter­actions. Inter­molecular N—H⋯O and O—H⋯O hydrogen bonds are also shown. Hydrogen bonds are shown as dashed lines.

The asymmetric unit of the title complex contains one water and two tetra­hydro­furane solvate mol­ecules. The water mol­ecules bridge the complex mol­ecules through N—H⋯O and O—H⋯S hydrogen bonds (Table 2 ▶) into centrosymmetric dimers arranged along the *c* axis, forming rings of graph-set 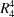
(12) (Fig. 3 ▶). In addition, classical O—H⋯O hydrogen bonds between tetra­hydro­furane and water mol­ecules and weak C—H⋯π inter­actions are observed (Table 2 ▶).

## Synthesis and crystallization   

Starting materials were commercially available and were used without further purification. The synthesis of the ligand was adapted from a procedure reported previously (Freund & Schander, 1902 ▶) and its structure is already published (de Oliveira *et al.*, 2014 ▶). 2-(1,2,3,4-Tetra­hydro­naphthalen-1-ylidene)-4-phenyl-hydrazinecarbo­thio­amide was dissolved in THF (2 mmol/40 ml) with stirring maintained for 30 min until the solution turned yellow. At the same time, a solution of nickel acetate tetra­hydrate (1 mmol/40 ml) in THF was prepared under continuous stirring. A mixture of both solutions was maintained with stirring at room temperature for 6 h. Crystals suitable for X-ray diffraction were obtained by the slow evaporation of the solvent.

## Refinement   

Crystal data, data collection and structure refinement details are summarized in Table 3 ▶. The imine and water H atoms were located in difference Fourier map, and were refined as riding with N—H = 0.88, O—H = 0.84 Å, and with *U*_iso_(H) = 1.2 *U*_eq_(N) or 1.5 *U*_eq_(O). All other H atoms were positioned with idealized geometry and refined using a riding model approximation, with C—H = 0.95-0.99 Å and with *U*_iso_(H) = 1.2 *U*_eq_(C). An outlier (17 0 20) was omitted in the last cycles of refinement.

## Supplementary Material

Crystal structure: contains datablock(s) I, publication_text. DOI: 10.1107/S1600536814016079/rz5128sup1.cif

Structure factors: contains datablock(s) I. DOI: 10.1107/S1600536814016079/rz5128Isup2.hkl

CCDC reference: 1013220

Additional supporting information:  crystallographic information; 3D view; checkCIF report

## Figures and Tables

**Table 1:** Selected bond lengths (Å)

Ni1—N2	1.9313 (14)	Ni1—S21	2.1524 (5)
Ni1—N22	1.9417 (14)	Ni1—S1	2.1664 (5)

**Table 2:** Hydrogen-bond geometry (Å, °) *Cg*1 and *Cg*2 are the centroids of the C32–C37 and C12–C17 rings, respectively.

*D*—H⋯*A*	*D*—H	H⋯*A*	*D*⋯*A*	*D*—H⋯*A*
N3—H1*N*⋯O1	0.88	2.06	2.934 (2)	172
N23—H2*N*⋯O51	0.88	2.02	2.895 (2)	171
O1—H1*O*1⋯S1^i^	0.84	2.63	3.4609 (16)	170
O1—H2*O*1⋯O41^ii^	0.84	2.00	2.836 (2)	173
C27—H27⋯*Cg*1^iii^	0.95	2.80	3.595 (2)	142
C54—H54*B*⋯*Cg*2^iv^	0.99	2.67	3.633 (2)	164

Symmetry codes: (i) 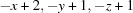; (ii) 
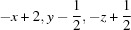
; (iii) 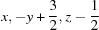; (iv) 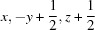.

**Table 3:** Experimental details

Crystal data	
Chemical formula	[Ni(C_16_H_16_N_3_S)_2_]·2C_4_H_8_O·H_2_O
*M*_r_	809.71
Crystal system, space group	Monoclinic, *P*2_1_/*c*
Temperature (K)	200
*a*, *b*, *c* (Å)	20.9248 (13), 8.7872 (5), 21.2833 (15)
β (°)	92.841 (8)
*V* (Å^3^)	3908.6 (4)
*Z*	4
Radiation type	Mo *K*α
μ (mm^−1^)	0.65
Crystal size (mm)	0.19 × 0.15 × 0.10

Data collection	
Diffractometer	Stoe *IPDS1*
Absorption correction	Numerical (*X-SHAPE* and *X-RED32*; Stoe & Cie, 2008 ▶)
*T*_min_, *T*_max_	0.787, 0.941
No. of measured, independent and observed [*I* > 2σ(*I*)] reflections	40358, 8412, 7107
*R*_int_	0.064
(sin θ/λ)_max_ (Å^−1^)	0.639

Refinement	
*R*[*F* ^2^ > 2σ(*F* ^2^)], *wR*(*F* ^2^), *S*	0.038, 0.090, 1.04
No. of reflections	8412
No. of parameters	488
H-atom treatment	H-atom parameters constrained
Δρ_max_, Δρ_min_ (e Å^−3^)	0.32, −0.48

Computer programs: *X-AREA* and *X-RED32* (Stoe & Cie, 2008 ▶), *SHELXS97* and *SHELXL97* (Sheldrick, 2008 ▶), *DIAMOND* (Brandenburg, 2006 ▶) and *publCIF* (Westrip, 2010 ▶).

## supplementary crystallographic information

### Crystal data

**Table:**

[Ni(C_16_H_16_N_3_S)_2_]·2C_4_H_8_O·H_2_O	*F*(000) = 1712
*M**_r_* = 809.71	*D*_x_ = 1.376 Mg m^−^^3^
Monoclinic, *P*2_1_/*c*	Mo *K*α radiation, λ = 0.71073 Å
Hall symbol: -P 2ybc	Cell parameters from 40358 reflections
*a* = 20.9248 (13) Å	θ = 2.5–27.0°
*b* = 8.7872 (5) Å	µ = 0.65 mm^−^^1^
*c* = 21.2833 (15) Å	*T* = 200 K
β = 92.841 (8)°	Prism, red
*V* = 3908.6 (4) Å^3^	0.19 × 0.15 × 0.10 mm
*Z* = 4	

### Data collection

**Table:**

Stoe IPDS-1 diffractometer	8412 independent reflections
Radiation source: fine-focus sealed tube, Stoe IPDS-1	7107 reflections with *I* > 2σ(*I*)
Graphite monochromator	*R*_int_ = 0.064
φ scans	θ_max_ = 27.0°, θ_min_ = 2.5°
Absorption correction: numerical (*X-SHAPE* and *X-RED32*; Stoe & Cie, 2008)	*h* = −26→26
*T*_min_ = 0.787, *T*_max_ = 0.941	*k* = −11→11
40358 measured reflections	*l* = −27→27

### Refinement

**Table:**

Refinement on *F*^2^	Secondary atom site location: difference Fourier map
Least-squares matrix: full	Hydrogen site location: inferred from neighbouring sites
*R*[*F*^2^ > 2σ(*F*^2^)] = 0.038	H-atom parameters constrained
*wR*(*F*^2^) = 0.090	*w* = 1/[σ^2^(*F*_o_^2^) + (0.0495*P*)^2^ + 1.531*P*] where *P* = (*F*_o_^2^ + 2*F*_c_^2^)/3
*S* = 1.04	(Δ/σ)_max_ = 0.002
8412 reflections	Δρ_max_ = 0.32 e Å^−^^3^
488 parameters	Δρ_min_ = −0.48 e Å^−^^3^
0 restraints	Extinction correction: *SHELXL97* (Sheldrick, 2008), Fc^*^=kFc[1+0.001xFc^2^λ^3^/sin(2θ)]^-1/4^
Primary atom site location: structure-invariant direct methods	Extinction coefficient: 0.0043 (6)

### Special details

**Table:**

Geometry. All e.s.d.'s (except the e.s.d. in the dihedral angle between two l.s. planes) are estimated using the full covariance matrix. The cell e.s.d.'s are taken into account individually in the estimation of e.s.d.'s in distances, angles and torsion angles; correlations between e.s.d.'s in cell parameters are only used when they are defined by crystal symmetry. An approximate (isotropic) treatment of cell e.s.d.'s is used for estimating e.s.d.'s involving l.s. planes.
Refinement. Refinement of *F*^2^ against ALL reflections. The weighted *R*-factor *wR* and goodness of fit *S* are based on *F*^2^, conventional *R*-factors *R* are based on *F*, with *F* set to zero for negative *F*^2^. The threshold expression of *F*^2^ > σ(*F*^2^) is used only for calculating *R*-factors(gt) *etc*. and is not relevant to the choice of reflections for refinement. *R*-factors based on *F*^2^ are statistically about twice as large as those based on *F*, and *R*- factors based on ALL data will be even larger.

### Fractional atomic coordinates and isotropic or equivalent isotropic displacement parameters (Å^2^)

**Table:**

	*x*	*y*	*z*	*U*_iso_*/*U*_eq_	
Ni1	0.763345 (10)	0.57702 (2)	0.542635 (10)	0.01599 (8)	
S1	0.85498 (2)	0.51110 (5)	0.50807 (2)	0.02150 (10)	
C1	0.84794 (8)	0.63429 (18)	0.44282 (8)	0.0176 (3)	
N1	0.80744 (7)	0.74626 (15)	0.43901 (6)	0.0193 (3)	
N2	0.77215 (7)	0.75911 (15)	0.49300 (6)	0.0176 (3)	
C2	0.74873 (8)	0.89550 (17)	0.50036 (8)	0.0176 (3)	
C3	0.75309 (9)	1.01216 (19)	0.44862 (8)	0.0239 (4)	
H3A	0.7289	0.9747	0.4106	0.029*	
H3B	0.7984	1.0230	0.4381	0.029*	
C4	0.72730 (9)	1.16776 (19)	0.46591 (9)	0.0272 (4)	
H4A	0.7214	1.2308	0.4275	0.033*	
H4B	0.7585	1.2197	0.4950	0.033*	
C5	0.66362 (10)	1.1516 (2)	0.49709 (10)	0.0312 (4)	
H5A	0.6470	1.2536	0.5076	0.037*	
H5B	0.6320	1.1016	0.4677	0.037*	
C6	0.67272 (9)	1.05810 (19)	0.55612 (9)	0.0249 (4)	
C7	0.64126 (10)	1.0929 (2)	0.61052 (11)	0.0339 (5)	
H7	0.6105	1.1724	0.6094	0.041*	
C8	0.65380 (11)	1.0144 (2)	0.66589 (11)	0.0380 (5)	
H8	0.6306	1.0376	0.7019	0.046*	
C9	0.70038 (11)	0.9014 (2)	0.66901 (10)	0.0334 (4)	
H9	0.7103	0.8502	0.7076	0.040*	
C10	0.73221 (9)	0.8639 (2)	0.61548 (8)	0.0249 (4)	
H10	0.7646	0.7880	0.6177	0.030*	
C11	0.71703 (8)	0.93683 (18)	0.55811 (8)	0.0204 (3)	
N3	0.88815 (7)	0.60673 (16)	0.39553 (7)	0.0219 (3)	
H1N	0.9185	0.5384	0.4020	0.026*	
C12	0.88886 (8)	0.67796 (19)	0.33607 (8)	0.0208 (3)	
C13	0.84043 (10)	0.7741 (2)	0.31185 (9)	0.0281 (4)	
H13	0.8049	0.7976	0.3362	0.034*	
C14	0.84464 (12)	0.8354 (2)	0.25151 (9)	0.0358 (5)	
H14	0.8116	0.9004	0.2351	0.043*	
C15	0.89587 (12)	0.8032 (2)	0.21546 (10)	0.0384 (5)	
H15	0.8985	0.8465	0.1748	0.046*	
C16	0.94352 (10)	0.7069 (3)	0.23938 (9)	0.0358 (5)	
H16	0.9787	0.6832	0.2146	0.043*	
C17	0.94061 (9)	0.6444 (2)	0.29920 (9)	0.0273 (4)	
H17	0.9738	0.5790	0.3150	0.033*	
S21	0.77040 (2)	0.40904 (5)	0.61626 (2)	0.02150 (10)	
C21	0.69687 (8)	0.45636 (18)	0.64762 (8)	0.0189 (3)	
N21	0.65424 (7)	0.54193 (16)	0.61843 (7)	0.0214 (3)	
N22	0.67275 (7)	0.58376 (15)	0.55827 (6)	0.0179 (3)	
C22	0.62487 (8)	0.63338 (18)	0.52238 (8)	0.0185 (3)	
C23	0.56144 (8)	0.6663 (2)	0.55078 (9)	0.0265 (4)	
H23A	0.5686	0.7398	0.5857	0.032*	
H23B	0.5451	0.5710	0.5689	0.032*	
C24	0.51066 (9)	0.7303 (2)	0.50437 (10)	0.0314 (4)	
H24A	0.4914	0.6466	0.4787	0.038*	
H24B	0.4763	0.7789	0.5276	0.038*	
C25	0.54023 (9)	0.8468 (2)	0.46163 (10)	0.0298 (4)	
H25A	0.5068	0.8908	0.4325	0.036*	
H25B	0.5599	0.9303	0.4871	0.036*	
C26	0.59041 (9)	0.7688 (2)	0.42476 (9)	0.0244 (4)	
C27	0.59580 (11)	0.7956 (2)	0.36072 (9)	0.0350 (4)	
H27	0.5691	0.8697	0.3402	0.042*	
C28	0.63934 (11)	0.7161 (3)	0.32660 (9)	0.0376 (5)	
H28	0.6429	0.7375	0.2832	0.045*	
C29	0.67788 (10)	0.6050 (2)	0.35546 (9)	0.0306 (4)	
H29	0.7070	0.5487	0.3317	0.037*	
C30	0.67348 (9)	0.57702 (19)	0.41940 (9)	0.0233 (4)	
H30	0.6994	0.5005	0.4392	0.028*	
C31	0.63103 (8)	0.66085 (18)	0.45479 (8)	0.0195 (3)	
N23	0.68631 (7)	0.39556 (17)	0.70534 (7)	0.0229 (3)	
H2N	0.7178	0.3384	0.7209	0.027*	
C32	0.62840 (9)	0.38489 (19)	0.73636 (8)	0.0221 (3)	
C33	0.57121 (9)	0.4550 (2)	0.71649 (9)	0.0285 (4)	
H33	0.5697	0.5192	0.6806	0.034*	
C34	0.51621 (10)	0.4305 (2)	0.74952 (10)	0.0339 (4)	
H34	0.4772	0.4776	0.7354	0.041*	
C35	0.51734 (10)	0.3391 (2)	0.80225 (10)	0.0356 (5)	
H35	0.4795	0.3227	0.8242	0.043*	
C36	0.57417 (11)	0.2718 (2)	0.82274 (10)	0.0357 (5)	
H36	0.5755	0.2098	0.8594	0.043*	
C37	0.62939 (10)	0.2937 (2)	0.79037 (9)	0.0298 (4)	
H37	0.6682	0.2464	0.8050	0.036*	
O1	0.99770 (7)	0.40290 (16)	0.41952 (8)	0.0417 (4)	
H1O1	1.0309	0.4309	0.4401	0.062*	
H2O1	1.0030	0.3084	0.4169	0.062*	
O41	0.97593 (8)	0.58636 (16)	0.08169 (9)	0.0448 (4)	
C41	0.90931 (12)	0.5585 (3)	0.07052 (16)	0.0538 (7)	
H41A	0.8912	0.6291	0.0381	0.065*	
H41B	0.8864	0.5735	0.1097	0.065*	
C42	0.90244 (13)	0.3977 (3)	0.04853 (15)	0.0531 (7)	
H42A	0.8981	0.3930	0.0020	0.064*	
H42B	0.8645	0.3492	0.0661	0.064*	
C43	0.96318 (13)	0.3203 (3)	0.07266 (12)	0.0455 (6)	
H43A	0.9842	0.2683	0.0380	0.055*	
H43B	0.9540	0.2443	0.1053	0.055*	
C44	1.00526 (12)	0.4463 (2)	0.10005 (13)	0.0448 (6)	
H44A	1.0089	0.4382	0.1465	0.054*	
H44B	1.0487	0.4394	0.0838	0.054*	
O51	0.78020 (7)	0.19400 (17)	0.76536 (7)	0.0344 (3)	
C51	0.81446 (12)	0.2516 (3)	0.82019 (11)	0.0467 (6)	
H51A	0.8107	0.1802	0.8558	0.056*	
H51B	0.7967	0.3512	0.8323	0.056*	
C52	0.88412 (11)	0.2691 (3)	0.80450 (11)	0.0408 (5)	
H52A	0.9120	0.2021	0.8314	0.049*	
H52B	0.8985	0.3758	0.8103	0.049*	
C53	0.88546 (10)	0.2222 (2)	0.73570 (10)	0.0347 (4)	
H53A	0.8835	0.3122	0.7077	0.042*	
H53B	0.9245	0.1630	0.7278	0.042*	
C54	0.82624 (9)	0.1255 (2)	0.72640 (10)	0.0297 (4)	
H54A	0.8106	0.1259	0.6817	0.036*	
H54B	0.8351	0.0192	0.7395	0.036*	

### Atomic displacement parameters (Å^2^)

**Table:**

	*U*^11^	*U*^22^	*U*^33^	*U*^12^	*U*^13^	*U*^23^
Ni1	0.01618 (12)	0.01577 (11)	0.01627 (11)	0.00208 (7)	0.00338 (8)	0.00228 (7)
S1	0.0184 (2)	0.0223 (2)	0.0243 (2)	0.00532 (15)	0.00491 (16)	0.00550 (15)
C1	0.0171 (8)	0.0168 (7)	0.0191 (7)	−0.0003 (6)	0.0028 (6)	−0.0024 (6)
N1	0.0229 (7)	0.0191 (6)	0.0164 (6)	0.0042 (5)	0.0067 (6)	−0.0001 (5)
N2	0.0176 (7)	0.0197 (6)	0.0157 (6)	0.0022 (5)	0.0035 (5)	−0.0018 (5)
C2	0.0175 (8)	0.0163 (7)	0.0189 (8)	0.0006 (6)	0.0019 (6)	−0.0006 (6)
C3	0.0301 (9)	0.0188 (8)	0.0234 (8)	0.0055 (7)	0.0056 (7)	0.0026 (6)
C4	0.0326 (10)	0.0158 (8)	0.0335 (10)	0.0048 (7)	0.0065 (8)	0.0019 (7)
C5	0.0275 (10)	0.0212 (8)	0.0451 (11)	0.0079 (7)	0.0031 (9)	−0.0016 (8)
C6	0.0211 (9)	0.0185 (8)	0.0358 (10)	−0.0011 (6)	0.0064 (8)	−0.0077 (7)
C7	0.0285 (10)	0.0273 (9)	0.0473 (12)	−0.0029 (7)	0.0167 (9)	−0.0147 (8)
C8	0.0411 (12)	0.0367 (11)	0.0384 (11)	−0.0119 (9)	0.0229 (10)	−0.0165 (9)
C9	0.0423 (12)	0.0348 (10)	0.0242 (9)	−0.0111 (8)	0.0117 (9)	−0.0071 (8)
C10	0.0308 (10)	0.0237 (8)	0.0204 (8)	−0.0057 (7)	0.0049 (7)	−0.0052 (7)
C11	0.0209 (8)	0.0191 (7)	0.0217 (8)	−0.0034 (6)	0.0062 (7)	−0.0051 (6)
N3	0.0213 (7)	0.0228 (7)	0.0220 (7)	0.0063 (6)	0.0054 (6)	−0.0005 (6)
C12	0.0224 (8)	0.0216 (8)	0.0187 (8)	−0.0039 (6)	0.0055 (7)	−0.0036 (6)
C13	0.0348 (10)	0.0295 (9)	0.0205 (8)	0.0045 (8)	0.0073 (8)	0.0007 (7)
C14	0.0518 (13)	0.0330 (10)	0.0229 (9)	0.0043 (9)	0.0053 (9)	0.0045 (8)
C15	0.0557 (14)	0.0387 (11)	0.0217 (9)	−0.0103 (10)	0.0102 (9)	0.0022 (8)
C16	0.0345 (11)	0.0485 (12)	0.0258 (10)	−0.0151 (9)	0.0146 (8)	−0.0088 (9)
C17	0.0224 (9)	0.0340 (9)	0.0261 (9)	−0.0048 (7)	0.0068 (7)	−0.0081 (7)
S21	0.0219 (2)	0.0233 (2)	0.0197 (2)	0.00565 (15)	0.00496 (16)	0.00627 (15)
C21	0.0214 (8)	0.0183 (7)	0.0171 (7)	−0.0010 (6)	0.0026 (6)	0.0006 (6)
N21	0.0214 (7)	0.0243 (7)	0.0189 (7)	0.0009 (6)	0.0060 (6)	0.0042 (5)
N22	0.0197 (7)	0.0178 (6)	0.0164 (6)	0.0016 (5)	0.0029 (5)	0.0011 (5)
C22	0.0192 (8)	0.0160 (7)	0.0204 (8)	0.0006 (6)	0.0022 (6)	−0.0015 (6)
C23	0.0189 (8)	0.0323 (9)	0.0288 (9)	0.0031 (7)	0.0042 (7)	0.0005 (7)
C24	0.0186 (9)	0.0361 (10)	0.0394 (11)	0.0043 (7)	0.0004 (8)	−0.0007 (8)
C25	0.0258 (10)	0.0294 (9)	0.0338 (10)	0.0105 (7)	−0.0030 (8)	0.0006 (8)
C26	0.0239 (9)	0.0239 (8)	0.0250 (9)	0.0041 (7)	−0.0035 (7)	−0.0011 (7)
C27	0.0404 (12)	0.0388 (11)	0.0251 (9)	0.0109 (9)	−0.0056 (8)	0.0050 (8)
C28	0.0486 (13)	0.0465 (12)	0.0175 (9)	0.0073 (10)	−0.0003 (8)	0.0013 (8)
C29	0.0337 (11)	0.0358 (10)	0.0225 (9)	0.0028 (8)	0.0034 (8)	−0.0079 (7)
C30	0.0245 (9)	0.0214 (8)	0.0238 (8)	0.0026 (7)	−0.0004 (7)	−0.0041 (6)
C31	0.0196 (8)	0.0192 (7)	0.0194 (8)	0.0008 (6)	−0.0015 (6)	−0.0029 (6)
N23	0.0228 (8)	0.0277 (7)	0.0185 (7)	0.0042 (6)	0.0039 (6)	0.0058 (6)
C32	0.0261 (9)	0.0223 (8)	0.0183 (8)	−0.0021 (6)	0.0064 (7)	−0.0010 (6)
C33	0.0273 (10)	0.0348 (10)	0.0238 (9)	0.0009 (8)	0.0056 (8)	0.0047 (7)
C34	0.0248 (10)	0.0429 (11)	0.0343 (11)	−0.0005 (8)	0.0068 (8)	0.0019 (8)
C35	0.0335 (11)	0.0394 (11)	0.0355 (11)	−0.0082 (9)	0.0170 (9)	0.0019 (9)
C36	0.0443 (12)	0.0347 (10)	0.0294 (10)	−0.0021 (9)	0.0153 (9)	0.0080 (8)
C37	0.0347 (11)	0.0301 (9)	0.0252 (9)	0.0038 (8)	0.0077 (8)	0.0063 (7)
O1	0.0317 (8)	0.0309 (7)	0.0613 (11)	0.0075 (6)	−0.0092 (7)	−0.0028 (7)
O41	0.0357 (9)	0.0262 (7)	0.0711 (12)	−0.0026 (6)	−0.0108 (8)	0.0095 (7)
C41	0.0352 (13)	0.0342 (11)	0.090 (2)	0.0029 (9)	−0.0133 (13)	−0.0017 (12)
C42	0.0458 (14)	0.0434 (13)	0.0685 (18)	−0.0082 (11)	−0.0121 (13)	−0.0022 (12)
C43	0.0606 (16)	0.0306 (10)	0.0443 (13)	0.0010 (10)	−0.0074 (11)	−0.0035 (9)
C44	0.0422 (13)	0.0310 (10)	0.0602 (15)	0.0027 (9)	−0.0069 (11)	0.0046 (10)
O51	0.0256 (7)	0.0425 (8)	0.0351 (8)	0.0066 (6)	0.0017 (6)	0.0013 (6)
C51	0.0449 (14)	0.0643 (15)	0.0309 (11)	0.0098 (12)	0.0022 (10)	−0.0047 (11)
C52	0.0393 (12)	0.0409 (11)	0.0409 (12)	0.0005 (9)	−0.0103 (10)	−0.0025 (9)
C53	0.0279 (10)	0.0370 (11)	0.0395 (11)	0.0015 (8)	0.0039 (9)	0.0030 (9)
C54	0.0296 (10)	0.0266 (9)	0.0329 (10)	0.0055 (7)	0.0006 (8)	0.0016 (7)

### Geometric parameters (Å, º)

**Table:**

Ni1—N2	1.9313 (14)	C24—H24B	0.9900
Ni1—N22	1.9417 (14)	C25—C26	1.506 (3)
Ni1—S21	2.1524 (5)	C25—H25A	0.9900
Ni1—S1	2.1664 (5)	C25—H25B	0.9900
S1—C1	1.7612 (17)	C26—C27	1.393 (3)
C1—N1	1.298 (2)	C26—C31	1.406 (2)
C1—N3	1.365 (2)	C27—C28	1.382 (3)
N1—N2	1.4008 (18)	C27—H27	0.9500
N2—C2	1.307 (2)	C28—C29	1.390 (3)
C2—C11	1.471 (2)	C28—H28	0.9500
C2—C3	1.511 (2)	C29—C30	1.390 (3)
C3—C4	1.522 (2)	C29—H29	0.9500
C3—H3A	0.9900	C30—C31	1.402 (2)
C3—H3B	0.9900	C30—H30	0.9500
C4—C5	1.524 (3)	N23—C32	1.412 (2)
C4—H4A	0.9900	N23—H2N	0.8800
C4—H4B	0.9900	C32—C33	1.393 (3)
C5—C6	1.505 (3)	C32—C37	1.401 (2)
C5—H5A	0.9900	C33—C34	1.395 (3)
C5—H5B	0.9900	C33—H33	0.9500
C6—C7	1.394 (3)	C34—C35	1.379 (3)
C6—C11	1.412 (2)	C34—H34	0.9500
C7—C8	1.379 (3)	C35—C36	1.379 (3)
C7—H7	0.9500	C35—H35	0.9500
C8—C9	1.390 (3)	C36—C37	1.388 (3)
C8—H8	0.9500	C36—H36	0.9500
C9—C10	1.388 (3)	C37—H37	0.9500
C9—H9	0.9500	O1—H1O1	0.8400
C10—C11	1.401 (3)	O1—H2O1	0.8397
C10—H10	0.9500	O41—C44	1.422 (3)
N3—C12	1.413 (2)	O41—C41	1.424 (3)
N3—H1N	0.8798	C41—C42	1.493 (3)
C12—C13	1.398 (3)	C41—H41A	0.9900
C12—C17	1.400 (2)	C41—H41B	0.9900
C13—C14	1.400 (3)	C42—C43	1.509 (4)
C13—H13	0.9500	C42—H42A	0.9900
C14—C15	1.378 (3)	C42—H42B	0.9900
C14—H14	0.9500	C43—C44	1.513 (3)
C15—C16	1.386 (3)	C43—H43A	0.9900
C15—H15	0.9500	C43—H43B	0.9900
C16—C17	1.391 (3)	C44—H44A	0.9900
C16—H16	0.9500	C44—H44B	0.9900
C17—H17	0.9500	O51—C51	1.431 (3)
S21—C21	1.7571 (17)	O51—C54	1.434 (2)
C21—N21	1.301 (2)	C51—C52	1.519 (3)
C21—N23	1.368 (2)	C51—H51A	0.9900
N21—N22	1.4050 (19)	C51—H51B	0.9900
N22—C22	1.304 (2)	C52—C53	1.523 (3)
C22—C31	1.470 (2)	C52—H52A	0.9900
C22—C23	1.513 (2)	C52—H52B	0.9900
C23—C24	1.522 (3)	C53—C54	1.508 (3)
C23—H23A	0.9900	C53—H53A	0.9900
C23—H23B	0.9900	C53—H53B	0.9900
C24—C25	1.521 (3)	C54—H54A	0.9900
C24—H24A	0.9900	C54—H54B	0.9900
			
N2—Ni1—N22	100.89 (6)	C26—C25—C24	108.67 (15)
N2—Ni1—S21	164.04 (5)	C26—C25—H25A	110.0
N22—Ni1—S21	85.90 (4)	C24—C25—H25A	110.0
N2—Ni1—S1	85.74 (4)	C26—C25—H25B	110.0
N22—Ni1—S1	162.63 (4)	C24—C25—H25B	110.0
S21—Ni1—S1	91.944 (18)	H25A—C25—H25B	108.3
C1—S1—Ni1	93.59 (5)	C27—C26—C31	118.83 (17)
N1—C1—N3	120.84 (15)	C27—C26—C25	121.67 (17)
N1—C1—S1	122.94 (12)	C31—C26—C25	119.43 (16)
N3—C1—S1	116.22 (12)	C28—C27—C26	121.06 (18)
C1—N1—N2	112.26 (13)	C28—C27—H27	119.5
C2—N2—N1	112.85 (13)	C26—C27—H27	119.5
C2—N2—Ni1	130.35 (11)	C27—C28—C29	120.40 (18)
N1—N2—Ni1	116.77 (10)	C27—C28—H28	119.8
N2—C2—C11	120.93 (14)	C29—C28—H28	119.8
N2—C2—C3	119.88 (14)	C28—C29—C30	119.46 (18)
C11—C2—C3	119.19 (14)	C28—C29—H29	120.3
C2—C3—C4	113.48 (14)	C30—C29—H29	120.3
C2—C3—H3A	108.9	C29—C30—C31	120.48 (17)
C4—C3—H3A	108.9	C29—C30—H30	119.8
C2—C3—H3B	108.9	C31—C30—H30	119.8
C4—C3—H3B	108.9	C30—C31—C26	119.68 (16)
H3A—C3—H3B	107.7	C30—C31—C22	121.87 (15)
C3—C4—C5	110.47 (15)	C26—C31—C22	118.36 (15)
C3—C4—H4A	109.6	C21—N23—C32	128.82 (16)
C5—C4—H4A	109.6	C21—N23—H2N	114.1
C3—C4—H4B	109.6	C32—N23—H2N	115.6
C5—C4—H4B	109.6	C33—C32—C37	118.67 (17)
H4A—C4—H4B	108.1	C33—C32—N23	124.97 (16)
C6—C5—C4	109.69 (16)	C37—C32—N23	116.34 (17)
C6—C5—H5A	109.7	C32—C33—C34	119.79 (18)
C4—C5—H5A	109.7	C32—C33—H33	120.1
C6—C5—H5B	109.7	C34—C33—H33	120.1
C4—C5—H5B	109.7	C35—C34—C33	121.2 (2)
H5A—C5—H5B	108.2	C35—C34—H34	119.4
C7—C6—C11	118.49 (18)	C33—C34—H34	119.4
C7—C6—C5	121.87 (17)	C34—C35—C36	119.13 (18)
C11—C6—C5	119.53 (16)	C34—C35—H35	120.4
C8—C7—C6	121.49 (19)	C36—C35—H35	120.4
C8—C7—H7	119.3	C35—C36—C37	120.63 (19)
C6—C7—H7	119.3	C35—C36—H36	119.7
C7—C8—C9	120.08 (18)	C37—C36—H36	119.7
C7—C8—H8	120.0	C36—C37—C32	120.52 (19)
C9—C8—H8	120.0	C36—C37—H37	119.7
C10—C9—C8	119.6 (2)	C32—C37—H37	119.7
C10—C9—H9	120.2	H1O1—O1—H2O1	102.5
C8—C9—H9	120.2	C44—O41—C41	107.61 (17)
C9—C10—C11	120.61 (18)	O41—C41—C42	107.1 (2)
C9—C10—H10	119.7	O41—C41—H41A	110.3
C11—C10—H10	119.7	C42—C41—H41A	110.3
C10—C11—C6	119.45 (16)	O41—C41—H41B	110.3
C10—C11—C2	121.53 (15)	C42—C41—H41B	110.3
C6—C11—C2	118.94 (16)	H41A—C41—H41B	108.6
C1—N3—C12	128.03 (15)	C41—C42—C43	104.7 (2)
C1—N3—H1N	118.2	C41—C42—H42A	110.8
C12—N3—H1N	113.8	C43—C42—H42A	110.8
C13—C12—C17	119.16 (17)	C41—C42—H42B	110.8
C13—C12—N3	123.95 (15)	C43—C42—H42B	110.8
C17—C12—N3	116.85 (16)	H42A—C42—H42B	108.9
C12—C13—C14	119.57 (18)	C42—C43—C44	105.43 (19)
C12—C13—H13	120.2	C42—C43—H43A	110.7
C14—C13—H13	120.2	C44—C43—H43A	110.7
C15—C14—C13	121.2 (2)	C42—C43—H43B	110.7
C15—C14—H14	119.4	C44—C43—H43B	110.7
C13—C14—H14	119.4	H43A—C43—H43B	108.8
C14—C15—C16	119.09 (19)	O41—C44—C43	107.00 (19)
C14—C15—H15	120.5	O41—C44—H44A	110.3
C16—C15—H15	120.5	C43—C44—H44A	110.3
C15—C16—C17	120.95 (18)	O41—C44—H44B	110.3
C15—C16—H16	119.5	C43—C44—H44B	110.3
C17—C16—H16	119.5	H44A—C44—H44B	108.6
C16—C17—C12	120.02 (19)	C51—O51—C54	107.23 (16)
C16—C17—H17	120.0	O51—C51—C52	107.68 (18)
C12—C17—H17	120.0	O51—C51—H51A	110.2
C21—S21—Ni1	94.84 (6)	C52—C51—H51A	110.2
N21—C21—N23	121.14 (15)	O51—C51—H51B	110.2
N21—C21—S21	123.24 (13)	C52—C51—H51B	110.2
N23—C21—S21	115.61 (13)	H51A—C51—H51B	108.5
C21—N21—N22	111.90 (14)	C51—C52—C53	104.34 (18)
C22—N22—N21	112.50 (14)	C51—C52—H52A	110.9
C22—N22—Ni1	129.64 (12)	C53—C52—H52A	110.9
N21—N22—Ni1	117.63 (11)	C51—C52—H52B	110.9
N22—C22—C31	121.71 (15)	C53—C52—H52B	110.9
N22—C22—C23	119.56 (15)	H52A—C52—H52B	108.9
C31—C22—C23	118.72 (15)	C54—C53—C52	102.98 (17)
C22—C23—C24	114.16 (15)	C54—C53—H53A	111.2
C22—C23—H23A	108.7	C52—C53—H53A	111.2
C24—C23—H23A	108.7	C54—C53—H53B	111.2
C22—C23—H23B	108.7	C52—C53—H53B	111.2
C24—C23—H23B	108.7	H53A—C53—H53B	109.1
H23A—C23—H23B	107.6	O51—C54—C53	105.00 (16)
C25—C24—C23	110.19 (16)	O51—C54—H54A	110.7
C25—C24—H24A	109.6	C53—C54—H54A	110.7
C23—C24—H24A	109.6	O51—C54—H54B	110.7
C25—C24—H24B	109.6	C53—C54—H54B	110.7
C23—C24—H24B	109.6	H54A—C54—H54B	108.8
H24A—C24—H24B	108.1		

### Hydrogen-bond geometry (Å, º)

*Cg*1 and *Cg*2 are the centroids of the C32–C37 and C12–C17 rings, respectively.

**Table:**

*D*—H···*A*	*D*—H	H···*A*	*D*···*A*	*D*—H···*A*
N3—H1*N*···O1	0.88	2.06	2.934 (2)	172
N23—H2*N*···O51	0.88	2.02	2.895 (2)	171
O1—H1*O*1···S1^i^	0.84	2.63	3.4609 (16)	170
O1—H2*O*1···O41^ii^	0.84	2.00	2.836 (2)	173
C27—H27···*Cg*1^iii^	0.95	2.80	3.595 (2)	142
C54—H54*B*···*Cg*2^iv^	0.99	2.67	3.633 (2)	164

Symmetry codes: (i) −*x*+2, −*y*+1, −*z*+1; (ii) −*x*+2, *y*−1/2, −*z*+1/2; (iii) *x*, −*y*+3/2, *z*−1/2; (iv) *x*, −*y*+1/2, *z*+1/2.
